# Learning experiences and identity development of Japanese nursing students through study abroad: a qualitative analysis

**DOI:** 10.5116/ijme.5e47.cf1b

**Published:** 2020-02-28

**Authors:** Jeffrey Huffman, Mami Inoue, Kiyomi Asahara, Michiko Oguro, Nobuko Okubo, Maki Umeda, Tomoko Nagai, Junko Tashiro, Kaoru Nakajima, Mari Uriuda, Aya Saitoh, Kana Shimoda

**Affiliations:** 1 Graduate School of Nursing Science, St. Luke's International University, Tokyo, Japan; 2 Chiba Faculty of Nursing, Tokyo Healthcare University, Chiba, Japan; 3 Research Institute of Nursing Care for People and Community, University of Hyogo, Kobe, Japan; 4 Center for International and Community Partnerships, St. Luke's International University, Tokyo, Japan; 5 Niigata University Graduate School of Medical and Dental Sciences, Niigata, Japan

**Keywords:** Study abroad, nursing education, identity, cultural competence, second language motivation

## Abstract

**Objectives:**

This study aimed to qualitatively analyze the experiences and perceptions of students at a nursing college in Japan who studied abroad in Asia and North America, thereby identifying the full range of benefits of study abroad programs for Japanese nursing students.

**Methods:**

We conducted a qualitative analysis of the reflection papers and free-response questionnaire items completed by 50 Japanese undergraduate nursing students who participated in 9 study abroad programs in Asia and North America. Content analysis of the data proceeded from typological and deductive to data-driven and inductive, recursively and collaboratively.

**Results:**

The results reveal perceived benefits in the areas of English language proficiency and motivation; knowledge of nursing practices, healthcare systems, and global health; cultural awareness and sensitivity; and various types of identity development (second-language motivation and identity, national/ethnic identity, professional identity, identity as a global citizen, and personal growth). It was also shown that students’ perceptions of what they learned or gained varied according to the specific characteristics of each study abroad program.

**Conclusions:**

Study abroad experiences are often critical turning points that enhance nursing students’ identity formation in the context of multiple and overlapping communities of practice. They also enhance core elements of the educational mission of a nursing college, particularly relating to liberal arts and internationalization. These findings can inform the development of assessment tools to be used in conjunction with study abroad programs at nursing colleges.

## Introduction

The resurgence of isolationism, jingoism, and anti-immigration sentiment notwithstanding, globalization is here to stay. The movement of people, ideas, information, and capital across borders will continue, and transnational interdependencies are not easily disentangled. Indeed, the world is experiencing improved communication technology, a shift to a globally-dispersed service-sector labor force, increasing ethnic pluralism in urban areas, and greater demographic mobility.^1^

The implication for higher education is a heightened recognition of the need for multilingual proficiency, cross-cultural awareness and sensitivity, and global networking and information sharing. In healthcare as well, a 2012 global independent commission found that a redesign of professional health education is needed “in view of the opportunities for mutual learning and joint solutions offered by global interdependence due to acceleration of flows of knowledge, technologies, and financing across borders”, and that “active student exchange can strengthen the bonds of empathy and solidarity that an interdependent but highly inequitable world so greatly needs”.^2^ To meet these needs, study abroad (SA) programs are becoming more common in nursing education, where a lack of culturally sensitive care is often cited as a continuing problem^3^ and the development of cultural competence is often the primary desired outcome.^4^

However, nursing students who study abroad reap benefits far beyond intercultural communication and sensitivity. Even a short-term experience can represent a “critical experience”^5^ that sparks further learning and growth. Furthermore, nearly all of the existing research on SA focuses on students from North America or Europe studying abroad in Europe.^6^^,^^7^ Non-Western perspectives are largely non-existent in the literature, particularly in the context of nursing education.

In this study, therefore, we qualitatively analyze the experiences and perceptions of students at a nursing college in Japan who studied abroad in Asia and North America. In addition to providing a rare window of insight into the experiences of Japanese nursing students studying abroad, our analysis yielded a framework of categories that is broader in scope than those in previous studies. The framework we propose (see Table 2) encompasses various types of identity transformation in addition to linguistic proficiency development, nursing and healthcare-related knowledge attainment, and increased cultural awareness and competence. We hope that researchers and educators at nursing colleges will make use of this framework to deepen their understanding of the benefits of SA.

### Definitions

For the purposes of this study, we define study abroad as any educational experience which involves a person (usually a student) leaving one’s home country for a definite period of time. Such experiences may be arranged and offered by a university, or an individual student may plan and engage in such an experience of their own initiative; however, only the former are investigated in this study. Experiences may be short term (1-4 weeks) or long term (4+ weeks), and they may involve traveling to developed or developing countries.^6^ They may also be designed to serve a variety of educational goals, from language proficiency improvement to cultural awareness and sensitivity, to critical and comparative observation of nursing practices and health policies in other countries, and beyond.

### Literature review: study abroad in nursing education

The literature on SA in nursing education coalesces around outcomes in the areas of cultural competence; knowledge of healthcare systems in other countries and global health in general; and personal development. A systematic review by Kulbok et al. found that knowledge and appreciation of cultural differences was one of the most common goals of SA programs for nursing students.^8^ Edmonds’ review noted that cultural competence, intercultural sensitivity, cultural self-efficacy, and cultural awareness were common themes throughout the literature.^9^ Koskinen and Tossavainen described a three-stage process of learning cultural competence, and also noted that the attitude and worldview of each participant greatly affects the learning outcomes,^10^ which is similar to Kinginger’s findings about study abroad in general.^7^ St. Clair and McKenry found that even short cultural immersion experiences resulted in significant differences in cultural self-efficacy, helped students overcome their ethnocentrism, and enhanced their cultural awareness and sensitivity.^11^ Greatrex-White concluded that such experiences triggered a positive “disturbance of the usual” which challenged students’ beliefs and assumptions.^12^

The Kulbok et al. review notes that while learning about healthcare systems, health issues, and nursing practices in other countries is often stated as a goal of SA programs for nursing students, this area receives little attention when reporting outcomes of such programs.^8^ A review by Button et al., however, found that students attain insights about the host country’s healthcare system and thereby notice strengths and weaknesses in their own country’s system.^6^ Kokko’s review describes many examples of participants identifying nursing practices in the host country that they approved or disapproved of, and their desire to bring effective practices back to their home country.^13^ Maltby et al. found that American students who studied in Bangladesh and the Netherlands learned about healthcare practices, the healthcare system, and social determinants of health.^14^ Evanson and Zust describe outcomes from a service-oriented trip to a developing country, which include students’ heightened awareness of social justice issues such as global health disparity, as well as their resulting desire to become advocates for political change.^15^^,^^16^

Many studies also report outcomes in the area of personal growth. Zorn’s International Education Survey showed that study abroad positively impacted students in the area of personal development.^17^ Kelleher’s review points out that such experiences can build self-confidence and self-reliance through overcoming challenges,^18^ and Kokko’s review notes that students often describe a feeling of “being a different person” after these experiences and that this may indicate an evolution in their perception of self in relation to the surrounding world.^13^ Green et al. note that students’ confidence was bolstered via the “untapping of previously untested inner resourcefulness”.^19^ The students in a study by Hoffart et al., which focused on cooperative (clinical) education experiences, also reported personal growth-oriented outcomes such as maturation, confidence and resilience development, and open-mindedness.^20^

### Literature review: communities of practice and identity development

Identity development is rarely mentioned explicitly in the literature on SA programs in nursing education; however, as we analyzed our own students’ data, we found identity development to be a prominent theme. Wenger’s social theory of learning and “communities of practice” is an applicable theoretical framework in this context.^21^ Wenger begins from the premise that humans are social beings, that “knowing” is a fundamentally social pursuit, and that meaningful engagement with the world is the ultimate goal of learning. Humans therefore construct their identities in relation to membership in “communities of practice”. Each community of practice that we belong to, whether defined by cultural, professional, or academic pursuits, develops its own routines, stories, and language, all of which is based on mutual engagement and the pursuit of common goals. In this view, identity is a dynamic and negotiated phenomenon that arises in the context of social connections and overlapping memberships. Andrew, Tolson, and Ferguson discussed the application of communities of practice to nursing,^22^ and Jorgensen and Hadders noted three overlapping communities of practice that were in play during a group of Norwegian nursing students’ clinical placement in Bangladesh.^23^

In the modern world of globalization, individualization, and social networking, identity is now viewed as “an open-ended and reflexive process of self-formation”, where we lead

lives that are “increasingly decision-dependent and in need of justification, re-elaboration, reworking and, above all, reinvention”.^24^ College students in particular are in the process of becoming who they will be, of “finding themselves”, to borrow an old cliché. In this context, SA experiences can be seen as “critical events” in identity development, which Layder describes as “non-routine and often unpredictable” events that require “substantial modifications of self-identity to enable the person to adjust to the changed circumstances”.^25^

Analyzing the lived experiences of the Japanese nursing students from this perspective heightened our awareness of outcomes in areas that we might not otherwise have noticed, such as second language identity and motivation, self-identification as a global citizen, identity as a Japanese person, and identity as a nurse.

In consideration of the existing literature and the gaps therein, which have been developed in this section, our aim in this study was to qualitatively analyze the experiences and perceptions of students at a nursing college in Japan who studied abroad in Asia and North America. This analysis yielded a more comprehensive understanding than has previously been reported regarding the learning and growth that occurs when nursing students study abroad.

## Methods

### Study design and participants

The participants in this study were 50 undergraduate students at a nursing college in Tokyo, Japan. The university offers 10 SA programs at eight universities in North America and Asia. The data analyzed in this study consisted of post-program reflection papers and free-response questionnaire items submitted by 50 of the 57 students who participated in 9 of these programs during the period from July 2015 through August 2016. The sample, therefore, consisted of all participating students who submitted the reflection papers or questionnaires. Permission for this study was obtained from the Ethics Committee of St. Luke’s International University (Approval #16-A081).

### Data collection

The reflection papers were free form, simply prompting students to write about what they had learned from the experience. For two of the nine programs, however, questionnaires were used instead of a free-form reflection paper. The questionnaires prompted students to comment on the following aspects of the programs: the content and structure of the program, difficulty level of the program, the English language classes that were part of the program, any change in their own language ability, aspects of their daily life during the program, the cultural activities that were part of the program, how their cultural understanding and knowledge of nursing had changed, and overall comments. Both the reflection papers and questionnaires were administered and collected by the Center for International and Community Partnerships between one week and two months after returning from the programs.

### Setting

The participants in this study were undergraduate students at a nursing college in Tokyo, Japan who participated in one of nine study abroad programs offered by the college. An overview of the programs, including the destination, objectives, and duration of each program, is provided in Table 1. Program participants attended orientation sessions and prepared presentations in English about topics such as their school, nursing education in Japan, and healthcare issues in Japan. After the program, they wrote reflective reports and gave English presentations about their experiences.

### Data analysis

This study focused on qualitative data analysis of reflection papers and free-response questionnaire items. The participants’ free responses on the questionnaires for these two programs, along with the reflection papers from the other seven programs, were read, coded, and analyzed using the following procedures.

**Table 1:** Overview of study abroad programs

Program	Objectives	Grade level of participants	Country	Duration
University of Illinois at Chicago (practicum)	Cultivate global outlook by taking U.S. nursing courses	4^th ^year	U.S.A.	8 weeks
Duke University (global & community health program)	Learn about community health issues from a global perspective	4^th^ year	U.S.A.	3 weeks
International Nursing Experience in Manila (practicum)	Nursing practicum on health issues in a developing country	4^th^ year	Philippines	10 days
Mahidol University (exchange)	Learn about the healthcare system and nursing practices in Thailand	2^nd^–4^th^ year	Thailand	2 weeks
Yonsei University (exchange)	Learn about the healthcare system and nursing practices in South Korea	2^nd^–4^th^ year	South Korea	2 weeks
Kaohsiung Medical University (exchange)	Learn about Chinese medicine and the healthcare situation in Taiwan	1^st^–2^nd^ year	Taiwan	2 weeks
Trinity University of Asia (service learning program)	Learn about volunteerism through a service learning experience	1^st^–3^rd^ year	Philippines	2 weeks
McGill University (medical EFL program)	Learn nursing and medical English in an applied, practical way	2^nd^–4^th^ year and Master’s	Canada	17 days
McGill University (English immersion program)	Intensive immersion program to learn English communication skills	1^st^-4^th^ year	Canada	3 weeks

Content analysis of the data followed the qualitative methodology outlined by Hatch, proceeding from typological and deductive to data-driven and inductive, in a recursive manner, until all researchers agreed that the resulting categories and subcategories were representative of the data.^26^ Initial categories arose from previous research and program objectives, and coding began in a deductive manner. Researchers then met regularly to discuss student responses which did not seem to fit into the pre-established categories, as well as categories that did not seem to reflect the data. Categories were thereby added, eliminated, and reorganized, and the resulting categories and subcategories are presented in Table 2.

## Results

The themes identified via the content analysis described above are presented in Table 2. The organization of our findings proceeds from ability acquisition (language and communication)  through knowledge acquisition (nursing-related, global health, and cultural) and identity development (cultural awareness; language identity and motivation; identification as a global citizen or a Japanese person or a nurse; personal growth).

**Table 2:** Final categories (themes) resulting from data analysis

Main categories	Subcategories
Language proficiency improvement	English communication ability improvement (general and nursing/medical)
	English presentation ability improvement
	English vocabulary/grammar (general and nursing/medical)
	English pronunciation/intonation improvement
	English listening ability improvement and enhanced sensitivity to a variety of English accents
	English group discussion ability
Learning about nursing, healthcare systems, society and culture	Learning about health issues and healthcare systems
	Learning about and experiencing nursing practice and education
	Learning about nursing/medical-related scientific research methodology
	Learning about other cultures and societies
Identity formation	Cultural sensitivity/competence
	Identity as an English speaker
	Identity as a Japanese person
	Identity as a nurse
	Identity as a global citizen
	Personal growth (independence, cooperativeness, broadening of worldview, confidence)

Students’ observations varied according to the specific characteristics of each program, as shown in Table 3. For example, students attending programs at North American universities, particularly those with English proficiency improvement as an explicit focus, mentioned language proficiency gains more often than programs in Asia that focused on visits to medical facilities.

**Table 3:** Comment frequency by category and program

Main categories	Program								
	McGill (medical EFL)	McGill (EFL immersion)	Mahidol	Yonsei	Kaohsiung	Trinity	Manila	Illinois	Duke
	n=6	n=11	n=4	n=4	n=9	n=5	n=6	n=4	n=3
Language proficiency improvement	^***^	^***^	^*^	^*^	^*^	^*^	^*^	^**^	^**^
Learning about nursing, healthcare systems, society and culture	^***^	^*^	^**^	^***^	^***^	^***^	^***^	^***^	^***^
Identity formation	^***^	^***^	^***^	^**^	^***^	^***^	^**^	^***^	^***^

*2 or fewer incidences; **3–9 incidences; ***10 or more incidences

### Language proficiency improvement

In an increasingly globalized society, high expectations are placed on Japanese university students to improve their English proficiency. This is true in the field of nursing as well, particularly due to increasing numbers of non-Japanese patients, the continuing trend toward evidence-based nursing practice, and the need to stay abreast of research being conducted globally. Although a measurable degree of improvement in language proficiency is not expected during a two-week program, students’ self-perception of improvements related to language proficiency are summarized here. Language-related motivation and identity is discussed later.

Participants often articulated their initial nervousness about participating in English conversations abroad. However, through the language-focused McGill programs in particular, many participants noted that their English communicative ability increased due to successfully making themselves understood within group settings. Some students remarked that they had learned to ask questions and give opinions in a classroom setting, in their residence hall or homestay environment, and while exploring the city. They felt that the act of speaking itself had activated and improved their English skills. Others said that they encountered difficulties participating in group discussions at first, such as feeling at a loss as to how to interject and participate. After a few weeks, however, they realized that their efforts were bearing fruit. One participant in the McGill (Canada) program wrote:

“I tried to communicate with people in Canada as much as I could, and I noticed a huge improvement in my speaking and listening skills. Being in an English environment really brought me out of my shell. In the end, I lost my fear of speaking out in English.”

It is possible that students in the current study attained slight proficiency gains even in the Asian programs, but that these gains were not salient enough to warrant inclusion in their reports.

### Learning about nursing and health care in other countries

Study abroad programs are seen as an important way for Japanese nursing students to learn about health policies and nursing practices in other countries, as well as health disparity. Many participants reported that they observed gaps in health conditions between countries, and that they became aware of the differences in healthcare policies, systems, and practices that underlie these gaps. For example, one student who participated in a service learning program in the Philippines wrote:

“Having universal health coverage is critical in order to provide access to health care. Health care systems differ among countries, often depending on budget restrictions. That is determined by the economic power of that nation.”

Another student, who participated in a McGill (Canada) program, noted that the experience made her realize the strong points of healthcare practice in her own country:

“I learned that the skills required of nurses differs between Canada and Japan, because of differences in the healthcare system. This program taught me that the quality of healthcare practice in Japan matches that of other developed countries”.

### Identity: cultural knowledge, awareness, and sensitivity

In this section we first note students’ acquisition of knowledge about other countries, and then draw a line from that knowledge to heightened cultural awareness and sensitivity. Participants often wrote that they valued the opportunity to learn about the culture of another country, and some students also pointed out how the culture and history of a country had resulted in the current healthcare situation. In addition, observing and experiencing different ways of life broadened their worldview. For example, a student who participated in the Thailand program wrote:

“I was exposed to cultures other than that of Japan. I had never been to a foreign country, and I was hardly interested in other countries. However, this program exposed me to foreign cultures and made me aware that I had previously lived in such a narrow world”.

Participants wrote about how these experiences made them reconsider their preconceived notions and taught them the importance of understanding and respecting other cultures. Some students commented that even if they had known about a country before, experiencing it firsthand made it become real to them for the first time. Others were surprised at the similarities between themselves and the people they met abroad since they had previously thought of “foreigners” (non-Japanese) as being “different”. For example, one student who participated in the Taiwan program remarked:

“I used to think that foreigners and foreign countries were very different from Japan. I had this image that they were all more confident and positive than Japanese people like myself. However, after this experience…I realized that they worry about things and cry about things just like me. I know this should be obvious, but by experiencing it in person…it just became more real to me somehow”.

This seemingly paradoxical juxtaposition of noticing “differences” while at the same time discovering the universal “sameness” of humanity reflects the different types of intercultural sensitivity described by Koskinen and Tossavainen, two of which are “acceptance” (acknowledging and accepting and adapting to cultural difference) and “minimization” (finding the differences unimportant compared to the similarities).^10^

### Identity as an English speaker and a global citizen

Even more salient than participants’ self-perception of English proficiency improvement was their identity development related to English ability. This involved an increased motivation to improve their English ability and a heightened willingness or desire to communicate in English and become a member of the global community. Participants explained that studying abroad opened their eyes to both their inadequacies and their potential as English-speaking Japanese citizens. Others wrote about their discovery that poverty and public health issues are not limited by national borders and that it is not enough for Japan to focus only on the Japanese health system. They felt that Japanese nursing professionals have a lot to contribute to the global health conversation, but without strong enough English skills, they found it difficult to make their voice heard in that conversation. For example, one participant in the Thailand program said:

“Without knowledge of English, and without the confidence to speak up in group discussions, I felt very frustrated that I couldn’t express my opinions. I realized that it is not enough only to be able to express myself in my mother tongue, and that I need to learn to communicate in other languages too”.

### Identity as a Japanese person

Participants also reflected on how their experience had affected the development of their identity as Japanese people. They wrote about the need for deeper understanding of their own country’s culture, history and social systems, as well as the ability to articulate that knowledge. Some commented that students from other countries were quite familiar with their own country and explained it with confidence, while they themselves were frustrated with their inability to talk about Japan and Japanese culture fluently. Others highlighted the importance of seeing themselves as representatives of their own culture. For example, one student who participated in the McGill (Canada) program stated:

“Unless we have deeper knowledge of our own culture and social systems, it is not possible to gain real insights about the culture of others”.

This participant’s observation echoes Bennett’s comments on cultural self-awareness; namely, that without awareness of our own culture we cannot “distinguish between projecting our own categories of perception and accessing the alternate categories of a different culture,” leading us to behave in “unconsciously ethnocentric ways.”^27^

### Identity as a nurse

Identity and self-concept development is closely related to “communities of practice”,^21^ whether the community in question is a tight-knit community of religious believers or a locally- or globally-situated profession consisting of members that have a common set of skills, knowledge, practices, and goals. Nursing is certainly such a community, but nursing students may have varying and dynamic levels of commitment and identification with their profession. Many SA participants wrote that the programs were invaluable opportunities to think about their career options from a broader perspective. Some commented that their observation of healthcare facilities and discussions with nurses and nursing students from other countries gave them renewed motivation to become a nurse. One student who participated in the Thailand program remarked:

“While engaging in deep exchanges with nursing students from various countries, I reflected on my own thoughts about nursing and my future goals in nursing”.

Some of the literature regarding SA experiences of nursing students mentions outcomes in the area of personal and professional development, and reports by Edmonds as well as Walsh and DeJoseph focus on professional self-efficacy and awareness issues.^9^^,^^28^ The participants in the current study noted that studying abroad gave them opportunities to reflect on their identity and motivation to become a nurse, and they often connected this to their future career plans as well.

### Personal growth

Closely connected with professional development is the personal development that occurs during SA experiences. Many participants in the current study wrote that they learned how to act on their own initiative while identifying their own role in group contexts, how to express their own opinions with confidence, how to respect the opinions of others, and how to cooperate with each other. These replies suggest that the participants were aware that these experiences resulted in personal growth. For example, one student who participated in the McGill (Canada) program remarked:

“I faced the challenge of identifying my role in the group under circumstances that were not familiar to me. Finally, I was able to overcome this challenge by dealing face-to-face with the issue and cooperating with members of the group”.

Another, who participated in the Illinois (U.S.) program, commented:

“Usually, Japanese students shared fewer opinions than those from other countries, but I tried to convey my thoughts with confidence, even if only a little”.

## Discussion

Participants in the North American programs tended to make positive comments about perceived language proficiency development during the programs. This is especially true for the McGill programs, which specifically include language learning components. Churchill and DuFon reviewed the research on language proficiency development through SA programs.^29^ Most research in this area identifies positive outcomes in specific skills (literacy, listening, speaking, pronunciation, and pragmatic abilities). However, there are mixed results when SA programs are compared with home country-based immersion programs. Ultimately, program length, what students do during the program, and individual differences in student motivation and learning strategies make it difficult to make generalizations. However, some studies of short-term programs have found modest gains in listening, pronunciation, and pragmatics.

Across all of the programs, participants focused on what they learned about nursing practice and health care systems in other countries. These findings echo those of Kirkham et al., who noted their participants’ heightened social consciousness, based on an enhanced understanding of “the social forces influencing health and illness, particularly circumstances of poverty, education, and historical legacies.”^30^ They are also consistent with the discussion in Green et al. regarding students’ observations of differences in nursing practice and education they encountered during SA programs.^19^ They expressed a desire (usually unrealized) to correct poor practices while at the same time wanting to “bring home” practices that they found admirable.

Many participants discussed how their SA experience contributed to heightened transcultural awareness and sensitivity. Previous research provides hope that these lessons will affect how participants approach their future nursing practice. Evanson and Zust found that practicing nurses who had participated in a SA program two years earlier still placed high value on the cultural awareness and global perspective they had cultivated, and which they could not have attained in a classroom in their home country. They felt that this perspective had been integrated into their approach to nursing, helping them provide more culturally sensitive care to their patients.^16^

The SA experience seems to have served as a “wake-up call” for many of the participants, both in terms of English ability and engagement as a global citizen. The comments in this section are consistent with the findings of Benson et al., who view SA as a “potentially critical experience that opens up second language identities to change.”^31^ Second language proficiency allows one to essentially become a different person in different situations, but people also strive for coherence in their identity construction. The concept of “possible selves”^32^ also sheds light on how SA experiences may impact the way participants see themselves in relation to the world. How they construct their ideal and non-ideal possible selves is important not only in terms of language proficiency and communication skills, but also in terms of national identity and professional identity.

Students in this study also noted various areas of personal development that were affected by these experiences. Personal development can be viewed as another aspect of identity construction that is likely to occur in nursing students during experiences abroad. Other studies have also noted the increased self-confidence, self-awareness, self-reliance, leadership skills, and general “life skills” that seem to be integral to SA experiences.^6^^,^^18^

As a limitation of this study, it is important to note that for the two McGill programs, free-response data from questionnaires were used, while open-ended reflection papers were used for the other seven programs. All data coding followed the same procedures, explained in the Methods section. However, the questionnaires directed participants to address specific topics, such as English improvement, making it more likely that those participants would comment on those issues.

## Conclusions

These data and findings reveal that studying abroad yields a wide array of benefits and thus plays an essential role in nursing education. The personal growth and cultural and language-related identity development that occurs during these experiences is an important extension of the liberal arts and humanities aspect of a nurse’s education. And the professional education of future nurses is also directly enhanced by learning about global nursing practices and healthcare systems; improving language proficiency and cultural competence in ways that are directly relevant to their future career contexts; and enhancing the development of their identity as nurses. Participants become more aware of themselves, of the world, and of a wider array of possibilities for and ramifications of the interaction between self and world than they had previously considered.

This study adds to a growing body of literature describing the outcomes of SA experiences for nursing students, and it provides a previously-missing Japanese perspective. These findings will benefit nursing educators internationally in a number of ways. First, we hope that nursing schools will make use of the evidence presented here and in other studies to justify an increase in the number and variety of SA programs, as well as the financial resources allotted thereto. We also recommend that educators and administrators consider how program design can influence outcomes. Indeed, programs can and should be designed in ways that fit with a college’s curriculum and mission. For example, each SA program can be designed to address specific educational goals, such as language proficiency, cultural awareness, comparing healthcare systems and policies, or firsthand observation of nursing practices and medical facilities. We also hope that educators and researchers will use our findings as the basis for questionnaires to evaluate the outcomes of their own programs. Finally, we hope this article will inspire nursing colleges to consider the important role SA programs can play in achieving a college’s broader educational and societal mission, which is to raise up future nurses who are not only knowledgeable and technically capable, but also self-reliant, self-aware, culturally sensitive, globally-oriented caregivers and leaders.

### Conflict of Interest

The authors declare that they have no conflicts of interest.
